# 

**Published:** 1846-10

**Authors:** 


ARTICLE IX.

**OUR EXCHANGES.**

*The Buffalo Medical Journal and Monthly Review of Medical and Surgical Science*, now in its second volume, has been much enlarged and improved in its appearance. Its accomplished editor, AUSTIN FLINT, M.D., is indefatigable in his efforts to render it worthy of the patronage of the medical public, and his efforts are seconded by the labors of several scientific gentlemen of note in his vicinity. No. IV, Vol. II. for September, is before us, and is replete with valuable matter. We always hail the arrival of this periodical with pleasure.

*The Western Lancet and Medical Library*, edited by L. M. LAWSON, M. D., and published at Lexington, Ky. has also been much enlarged, and is issued bi-monthly. Additionally to the Journal there is appended a library department, in which a valuable work on Auscultation is in progress of publication, a portion being appended to each number.

### TO READERS AND CORRESPONDENTS.

Communications have been received from Prof. M'Lean, Drs. Jos. W. Cooke, Lyman Brackett, and Wm. Butterfield.

We have also received the following works and periodicals:—

The United States Dissector or Lessons in Practical Anatomy. By Wm. E. Horner, M. D. Edited by Henry H. Smith, M. D. Phil: Lea & Blanchard. 1846. pp. 444. (From the Publishers. For sale by Brautigam & Keen, Chicago.)

Coley on Infants and Children, in Select Medical Library. Edited by John Bell, M. D. Philadelphia: Ed. Barrington & Geo. D. Haswell. 1846. pp. 414. (From the Publishers.)

Researches, Historical, Topographical, and Critical, on Yellow Fever. By Bennet Dowler, M. D., of New Orleans.

A Review of "Homœpathy, Allopathy & Young Physic," by L. M. Lawson, M. D.

Summary of the Transactions of the College of Physicians of Philadelphia, from April to August, 1846.

Statements of Facts in Relation to the Expulsion of James C. Cross from Transylvania University.

A Vindication of Character and an examination of the Accusations of Dr. T. Reyburn's Supplement to the St. Louis Med. and Surg. Journal. By F. Knox, M. D,

Dr. Fougeaud's second defence against the charges of Dr. Reyburn.

Circular announcing a Course of Private Medical Instruction by Wm. H. Van Buren, M. D., and C. E. Isaacs, M. D., of New York City.

Report of the Medical Department of the University of Pennsylvania, for the year 1846: to the Alumni of the School. By the Medical Faculty.

Annual Circular of the Medical Department of Illinois College, Jacksonville, Ill.

Annual Announcement of the Medical Department of Pennsylvania College, Philadelphia.

Catalogue of the Faculty and Students of the Medical Department of the University of the State of Missouri for 1845 and '46.

Annual Circular of the Massachusetts Medical College, with a history of the Medical Department of Harvard University. A catalogue of Graduates, &c., Boston.

Annual Announcement of the Medical Department of the St. Louis University.

Catalogue of works in all branches of Medicine and Surgery, including the Collateral Sciences. Published by Ed. Barrington & Geo. D. Haswell, 293 Market st. Philadelphia.

I. & H. G. Langley's Medical Catalogue for 1846. No. 8 Astor House, New York.

The Journal of Health and Monthly Miscellany, Boston. (In Exchange.)
The Medical Examiner. (In Exchange.)
The Bulletin of Medical Science. (In Exchange.)
The Western Journal of Medicine & Surgery. (In Exchange.)
The Buffalo Journal, and Medical Review. (In Exchange.)
Southern Medical & Surgical Journal. (In Exchange.)
The Western Lancet & Medical Library. (In Exchange.)
The St. Louis Medical and Surgical Journal. (In Exchange.)
The Medical News and Library. (In Exchange.)
The American Journal and Library of Dental Science. (In Exchange.)
The Boston Medical and Surgical Journal. (In Exchange.)
The Missouri Medical & Surgical Journal. (In Exchange.)
The New York Medical & Surgical Reporter. (In Exchange.)
The New York Journal of Medicine and the Collateral Sciences. (In Exchange.)

---

### CONTRIBUTORS TO THE ILLINOIS & INDIANA MED. & SURG. JOUR.

A. H. Howland, M. D , Ottawa, Ill.
A. G. Henry, M. D., Pekin, Ill.
Edward Lewis, M. D., Jackson, Mich.
John McLean, M. D. Jackson, Mich.
Daniel Stahl, M. D., Quincy, Ill.
Edward Mead, M. D. Geneva, Ill.
E. Fisher, Waynesville, Ohio.
H. S. Huber, M. D., Chicago, Ill.
Samuel G. Armor, M. D., Rockford, Ill.
Ira C. Bachus, M. D., Jackson, Mich.
J. G. Cornell, M. D., Spring Arbor, Mich.
G. N. Fitch, M. D., Logansport, Ind.
John F. Henry, M. D., Burlington, Iowa.
E. Deming, M. D., Lafayette, Ind.
S. B. Thayer, Battle Creek, Mich.

